# Antibacterial and antioxidant double-layered nanofibrous mat promotes wound healing in diabetic rats

**DOI:** 10.1038/s41598-023-30240-8

**Published:** 2023-02-23

**Authors:** Fereshteh Nejaddehbashi, Zeinab Rafiee, Mahmoud Orazizadeh, Vahid Bayati, Aliasghar Hemmati, Mahmoud Hashemitabar, Pooyan Makvandi

**Affiliations:** 1 Cellular and Molecular Research Center, Medical Basic Sciences Institute, Ahvaz Jundishapur University of Medical Sciences, Ahvaz, Iran; 2 Department of Experimental Medical Science, Faculty of Medicine, Lund University, 22100 Lund, Sweden; 3 Wallenberg Centre for Molecular Medicine, Lund University, 22100 Lund, Sweden; 4 Department of Anatomical Sciences, School of Medicine, Ahvaz Jundishapur University of Medical Sciences, Ahvaz, Iran; 5 Marine Pharmaceutical Research Center, School of Pharmacy, Ahvaz Jundishapur University of Medical Sciences, Ahvaz, Iran; 6 Centre for Materials Interfaces, Istituto Italiano di Tecnologia, Viale Rinaldo Piaggio 34, 56025 Pontedera, Pisa, Italy; 7 Quzhou Affiliated Hospital of Wenzhou Medical University, Quzhou People’s Hospital, Quzhou, 324000 Zhejiang China

**Keywords:** Biological techniques, Cell biology, Structural biology

## Abstract

Diabetic wounds are problematic to heal owing to microbial infections as well as decreased proliferation and high concentrations of reactive oxygen species. In this study, a double-layered nanofibrous mat containing grape seed extract (GSE) and silver sulfadiazine (SSD) was fabricated. A synthetic biodegradable polymer, e.g., polycaprolactone (PCL), and a natural material (i.e., collagen) were employed as wound dressing substances. The results showed that GSE possesses antioxidant activity which can be helpful in reducing free radicals. The platform exhibited antibacterial activity against gram-positive and -negative bacteria. The double-layered nanofibrous mat containing GSE and SSD not only was not toxic but also amplified the cell proliferation compared to a pure mat, showing the effect of plant extract. After induction of a round wound, the animals were divided into three groups, namely (1) normal group (receiving + GSE/-GSE nanofiber), (2) diabetic group (receiving + GSE/-GSE nanofiber), and (3) control group (receiving gauze). In vivo evaluation demonstrated no significant differences in the healing process of normal rats. Surprisingly, fully repaired skin was observed on day 14 in the double-layered nanofibrous mat containing GSE in the normal and diabetic groups whereas the wound of diabetic rats treated with pure mat was not completely healed. The macroscopic and microscopic results after 14 days showed the following order in wound repair: Normal/ + GES > Diabetic/ + GSE > Normal/-GES > Diabetic/-GSE > control (with gauze) (*p* < 0.05). Accordingly, the double-layered nanofibrous mat containing GSE and SSD used in the present study could be considered as a suitable wound dressing in order to shorten healing time and prevent infection during the wound healing process.

## Introduction

Skin is the largest organ that covers the outer surface of the human body and plays a protective role against pathogens, microorganisms, and dehydration^1^. When the skin is damaged by an injury, a dynamic recovery process called wound healing is commenced, which improves the reformation of the injured skin. It includes hemostasis, inflammation, proliferation, and maturation of cells, recovering the damaged tissue after a series of nesting stages^2,3^. Overall, properly determined steps including hemostasis, inflammation, proliferation and remodeling are necessary for the progress of wound healing^4–7^. Chronic wounds pose critical problems for clinicians because they are found in a wide variety of patients and reduce the quality of life^8^.

One of the main concerns of clinicians in this regard is using the best material in the dressings used to cover these wounds. PCL, as a synthetic material with a wide range of advantages such as biocompatibility, biodegradability, nontoxicity, is approved by the Food and Drug Administration (FDA) to be employed for medical purposes. PCL has noticeable physical and mechanical properties in proper handling of wound areas. Despite its unique and useful characteristics, some features of this polymer such as its limited hydrophilicity leading to high fluid loss, low adhesion to wound bed, and delayed reepitelialization have been regarded as its main drawbacks^9^.

COLL is another well-studied natural structural protein within the extracellular matrix (ECM) of the skin with some advantages such as hydrophilicity and a faster degradation rate compared with PCL. Therefore, it can be blended with PCL to increase the hydrophilicity and degradation rate with desired fibrous structures and promote cell attachment and proliferation. COLL-PCL composite nanofibers have been reported to be promising candidates for tissue engineering applications. Combining both materials using electrospinning method can also lead to the formation of a nanofibrous structure similar to ECM microstructure. Cell surface receptors can thus easily recognize natural materials in the nanofibrous mat and attach to it. Composite polymers including natural and synthetic polymers have also demonstrated lower toxicity effects^10^.

Electrospinning is an important technique used to establish an integrative framework of nano-scale fibers. These nanofibers are definitely similar to native ECM that promote cell adhesion and proliferation. Organized conformation provides a broad surface zone to volume ratio along with suitable porosity of electrospun framework with small-sized pores that ameliorate the hemostasis at the local wound site instead of using the specific drugs^11^.

Owing to the structural characteristics of an electrospun network of nanofibers, these fibers are capable of absorbing exudates and providing wettability microenvironment suitable for cell respiration and proliferation. For example, very small size pores seem to reduce the possibility of bacterial infection and allow high permeability, preventing the injured area from dehydration. Another significant privilege of this technique is its capability and plasticity in loading drugs and some other crucial biomolecules such as growth factors, nanoparticles, antimicrobials, and anti-inflammatory reagents into the nanofibers^12^.

Infection in wounds leads to increased amount of exudates, preventing formation of granulation tissue and delaying the wound healing process. It is necessary to use healthy substances to treat wounds. Some biopolymers are routinely used to create healthy materials with wound healing capabilities^13^. Due to the formation of biofilm, wounds and burns are likely to be accompanied with chronic infections caused by bacteria such as *Staphylococcus aureus* and *Pseudomonas aeruginosa*. Thus, the presence of an effective antibacterial in wound dressings seems necessary. Silversulfadiazine has been used as an external antimicrobial agent since 1960, and it is used in wounds and burns with partial and full thickness of the skin to prevent infection. Silversulfadiazine, however, has a series of side effects such as allergic reactions and reduced white blood cells. Moreover, whether silversulfadiazine will actually lead to wound healing or whether it has genuine antibacterial properties has been a matter of dispute. Therefore, to alleviate these limitations, nanotechnology has placed silver sulfadiazine in nanofibrous scaffolds^14^.

There are many different approaches to wound healing, all of which need a careful balance of oxidative stress and antioxidants, which can help to reduce oxidative stress at the wound and increasingly accelerate the healing process^15^. Grape seed extract (GSE), which contains most of the active substances and a mixture of polyphenols, has shown the potential to scavenge free radicals, being 20–50 times more efficient than vitamin E or C in wound healing. Epicatechin and Catechin are two of the most essential biochemical components of GSE. Therefore, it appears that GSE affects the wound healing process through two routes, namely the repairing of injured blood vessels and promoting the process of defensive cell aggregation at the wound site, significantly evacuating the site from bacterial infections^16,17^.

Previous investigations have shown that electrospun COLL-based nanofibrous mats can improve wound healing in vitro and in vivo. In the present study, we used a natural polymer (COLL), a biodegradable synthetic polymer (PCL) along with SSD as an effective antiinfection substance in synergistic effect with GSE as an antimicrobial agent against different bacterial, viral, and fungal pathogens**.** To obtain a cost-effective and optimized nanofibrous mat with antibacterial efficiency for wound healing, a composite of PCL/COLL with SSD and GSE was selected to fabricate the electrospun mat. The main purpose of this study was to develop antimicrobial wound dressing capable of cell seeding.

In the present study, we fabricated a biocomposite double-layered nanofibrous mat based on polycaprolactone (PCL)/collagen and PCL/silver sulfadiazine (SSD) containing GSE as a wound dressing platform. After physicochemical characterization, antibacterial and antioxidant activity along with MTT assay were evaluated. Finally, in vivo evaluation of the healing ability in normal and diabetic rat models was carried out.

## Materials and methods

### Materials

Streptozotocin (STZ), poly (ε-caprolactone, Mw of 80 kDa), MTT (M2128), dialysis bag (12 and 100 KDa), and collagen type I (C9791) were obtained from Sigma Aldrich (St. Louis, MO, USA), and acetic acid (purity 99.8%) was prepared from Merck (Darmstadt, Germany). Fetal bovine serum (FBS), PBS, DMEM/ F12, and trypsin were acquired from Gibco (Grand Island, NY, USA), and silver sulfadiazine (SSD) was purchased from Sinadaru (Tehran, Iran). Tolou Gostar Bokhara Co. donated the grape seed extract powder (GSE) (Ahvaz, Iran).

### Fabrication of the PCL/COLL nanofibers

PCL with a concentration of 15% wt/v and collagen with a concentration of 1% wt/v were dissolved in 90% acetic acid and prepared at room temperature^18^. The COLL solution was mixed with the PCL solution in a 30:70 weight ratio and stirred for 24 h. The solution was transferred into a 10 ml syringe, and electrospinning process was carried out at ambient temperature under the following conditions: 125 rpm, 16 cm distance, 17kv voltage and 0.5 ml/h injection rate. The collected nanofibers on aluminum foil were dried overnight, then the nanofibrous mat was detached and utilized for the other tests and experiments.

### Fabrication of the PCL/SSD nanofibers

Designing this layer of the nanofibrous mat was done according to a previous study, with some modification^19,20^. In brief, PCL pellets were dissolved in 90% acetic acid to prepare 15% wt./v PCL solution to which 3 mg/mL SSD was then added. The solutions were mixed with a magnetic stirrer overnight**.** The electrospinning condition was as follows: 125 rpm, 20 kv, 18 cm distance, and 0.5 ml/h injection rate.

### Preparation of the double-layered nanofibrous mat (PCL/Coll, PCL/SSD) with GSE

PCL/SSD was subjected to electrospinning as the first layer. In the next step, the PCL/Coll composite was put on the electrospinning system, and the second layer was covered with PCL/Coll fibers. Then the double-layered nanofibrous mat was immersed in GSE solution (2% Wt) overnight until the color of the scaffold changed to brown.

### Characterization

Surface morphology of the electrospun nanofibers was examined under a Field-Emission Electron Microscope (Mira3Tescan, Czech). Also, the morphological features of the edges of fibers formed with PCL/SSD and PCL/Coll were studied. Qualitative elemental analysis such as the presence of SSD and GSE loaded into nanofibers was also performed with EDX.

Thermal behavior of PCL/SSD, PCL/Coll, and the double-layered nanofibrous mat was analyzed based on Thermogravimetric analysis (TGA) from room temperature to 600˚C at a heating rate of 10˚C/minute (STA503, Germany) under N2 flow.

Tensile tests were carried out to evaluate the mechanical properties of the monolayered and double-layered nanofibrous mats. The nanofibrous mat was cut in rectangular shapes (2 × 5 cm). The film thickness was then measured using a thickness gauge, and the tensile test was carried out on a universal tensile machine (INSTRON 5967 USA) equipped with a 60 N load cell at 5 mm/minute speed until the samples ruptured.

### In vitro release study

To determine the GSE release, the nanofibrous mat containing GSE (average weight 50 mg) was placed in a 50‐mL centrifuge tube in a dialysis bag (12KDa), immersed in 40 mL of PBS (pH:7.4), and incubated at 37 °C in a continuous horizontal shaker. At predetermined time points (0.5, 1, 2, 3, 4, 5 h, 1 day, and 2 days) 2 mL of the solution was removed and replaced with 2 mL of fresh PBS. The release profile of GSE was determined using atomic absorption spectrophotometer (Shimadzu model UV‐1700, Columbia, MD, USA) at the 270 nm. After that, the data were statistically analyzed, and the results were expressed in mean and standard deviation. The findings were obtained using distilled water as a blank. The calibration curve was plotted using the x-axis for drug concentration and the y-axis for absorbance.

### Antioxidant activities

The antioxidant activities of the GSE extract were evaluated using DPPH radical scavenging activity^21^.GSE was dissolved in ethanol to obtain a stock solution (250 µg/mL. The extracts were prepared by five-time dilution method in 96-well microtitre plates (200, 100, 50, 25, 12.5, 6.25 µg/ml). DPPH radical solution was prepared for 200 μM with 95% ethanol.

An aliquot of extract (10 μL) was mixed to 195 μL of methanolic DPPH in 96-well microtitre plates. The reaction mixtures were incubated at room temperature for 30 min in the dark.

Absorbance was measured at 517 nm by Microplate Reader.

The free radical scavenging activity was calculated as follows:

$$\% \;{\text{inhibition}} = \left\{ {\left( {{\text{Ab}}\;{\text{control}} - {\text{Absample}}} \right)/\left( {{\text{Ab}}\;{\text{control}}} \right)} \right\} \times {1}00$$

Ab control = absorbance of DPPH alone.

Ab sample = absorbance of DPPH along with different concentrations of extracts.

The lower absorbance of the reaction mixture indicated higher DPPH radical scavenging activity**.**

### Antibacterial tests

The inhibition zone test was used to evaluate the antibacterial properties of the double-layered nanofibrous mat containing SSD against *Staphylococcus aureus* (ST ATCC29213) and *Pseudomonas aeruginosa* (PS ATCC27853)^20,22^.

### Cell viability and adhesion studies

FE-SEM was used to examine cell attachment and morphology of HDF stem cells on a double-layered nanofibrous mat with and without GSE. Both nanofibers were cut out with a punch and sterilized via UV radiation for 1 h before being placed in 96-well cell culture plates. The HDFs were acquired according to the serial culture plate method similar to previous procedures^23^. The cells were then seeded on each nanofiber with 8000 cells/cm^2^ and incubated at 37˚C for 2 days. After 2 days, the fibers were fixed with 4% paraformaldehyde for 1 h at 4˚C and then dehydrated in a series of ethanol and allowed to air-dry overnight. After complete drying, nanofibers with cells were sent to BuAli Research Institute at Mashhad University of Medical Sciences and observed using FE-SEM.

The MTT assay (3- (4, 5-dimethylthiazol-2-yl)-2, 5- diphenyl tetrazolium bromide) was used to determine cell viability and proliferation, according to the manufacturer’s (Invitrogen) instructions. In brief, the medium was removed 24, 48, and 72 h after cell seeding, and MTT reagent was added. Then the cells were incubated for 4 h in an incubator at 37 °C and with 5% CO_2_ until formazan crystals formed. After the crystals were dissolved in DMSO, each homogenate sample was transferred in triplicate to a 96-well plate. The final measurement was taken at 570 nm with a microplate reader (Bio-Rad 680, USA). The data were analyzed using GraphPad Prism software's paired t-test analysis**.** (Sign test, *p* < 0.05).

### In vitro cell migration assay

An in vitro wound-healing assay was used to evaluate the wound healing potential of the realized formulations^24^. To this aim, fibroblast cells^23^ were seeded at a density of 4 × 10^4^ cells/ml on six-well plates to obtain a monolayer of cells. Then, a scratch was made across the middle of each well using a sterile 1000 μL pipet tip, and the plates were washed twice with PBS to remove the detached cells. A new medium was added, and approximately 1 × 1 cm sized of the prepared scaffolds were placed over the scratched area. Scratches were observed and imaged under the microscope (Olympus IX71) immediately after the wounding procedure and after 24 and 48 h of incubation.

### In vivo evaluation

#### Ethics declarations

Animal experiments were performed according to ethical guidelines and approved by the Ethics Committee of Ahvaz Jundishapur University of Medical Sciences (IR.AJUMS.ABHC.REC.1400.023). All methods were reported in accordance with ARRIVE guidelines (https://arriveguidelines.org).

#### Study design

The effect of designed nanofibrous mat was evaluated on 27 male Sprague–Dawley rats. Three groups of rats were used in this study: 1. The normal rat group (n = 9) receiving double-layered nanofibrous mats with and without GSE; 2. Diabetic rat group (n = 9) receiving double-layered nanofibrous mats with and without GSE. 3. Control group (n = 9) including rats receiving gauze at their wound site. The animals were euthanized on days 3, 7, and 14, and the wound closure process were documented using a digital camera.

For evaluation of the prepared nanofibrous mats as proper diabetic wound dressing, streptozotocin (50 mg/kg)^25^ was administered intraperitoneal in the rats to induce diabetic mellitus (DM), and blood glucose levels were monitored daily and kept between 400 and 500 mg/dl. Then a bilateral full-thickness wound (100 mm^2^) was created on the dorsal skin of male Sprague–Dawley rats (250 g), which were kept in standard conditions with 12/12-h light/dark cycles at a regulated temperature (21 ± 2˚C). The rats were anesthetized with 40 mg/kg ketamine and 5 mg/kg xylazine prior to the creation of wounds. The skin was then shaved with an electric clipper, and the dorsal skin was sterilized with 70% ethanol before a scissor wound was created from behind along the dorsal side of the skin. After implantation of scaffolds with a thickness of 500 µm, 5–0 nylon sutures were used to stabilize the scaffolds.

#### Histological analysis

For histological analysis, the autopsy was obtained from surrounding skin including the wound area, the specimens were fixed with 10% formalin solution and embedding in paraffin for histological analysis. A rotating microtome was used to cut samples into 5-µm thick sections for histological studies and to assess the wound area and repair process. Masson's trichrome and hematoxylin / eosin (H&E) staining method was used.

### Statistical analysis

All results are presented as mean ± standard deviation. The difference between two sets of data was assessed using one‐way analysis of variance (ANOVA) followed by Tukey post hoc analysis. Each experiment was repeated at least three times.* P* < 0.05 was considered statistically significant (Minitab Express TM Version 1.4.0).

## Results

### Fabrication and characterization

**Figure 1:**
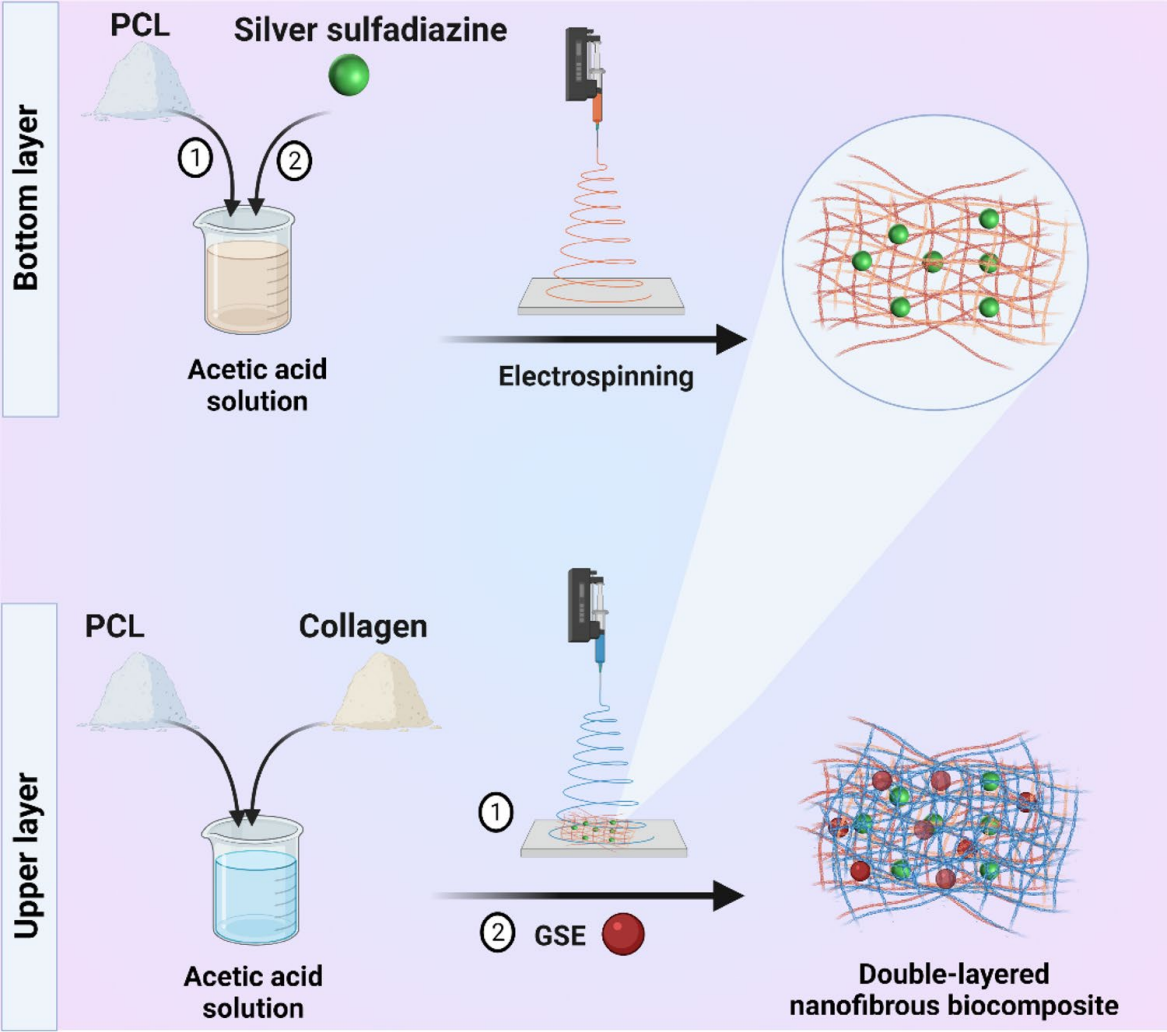
Manufacturing process of the scaffold via electrospining. Schematic illustration of fabrication of the double-layered nanofibrous mat.

A schematic illustration of the double-layered nanofibrous mat is shown in Fig. 1.

**Figure 2:**
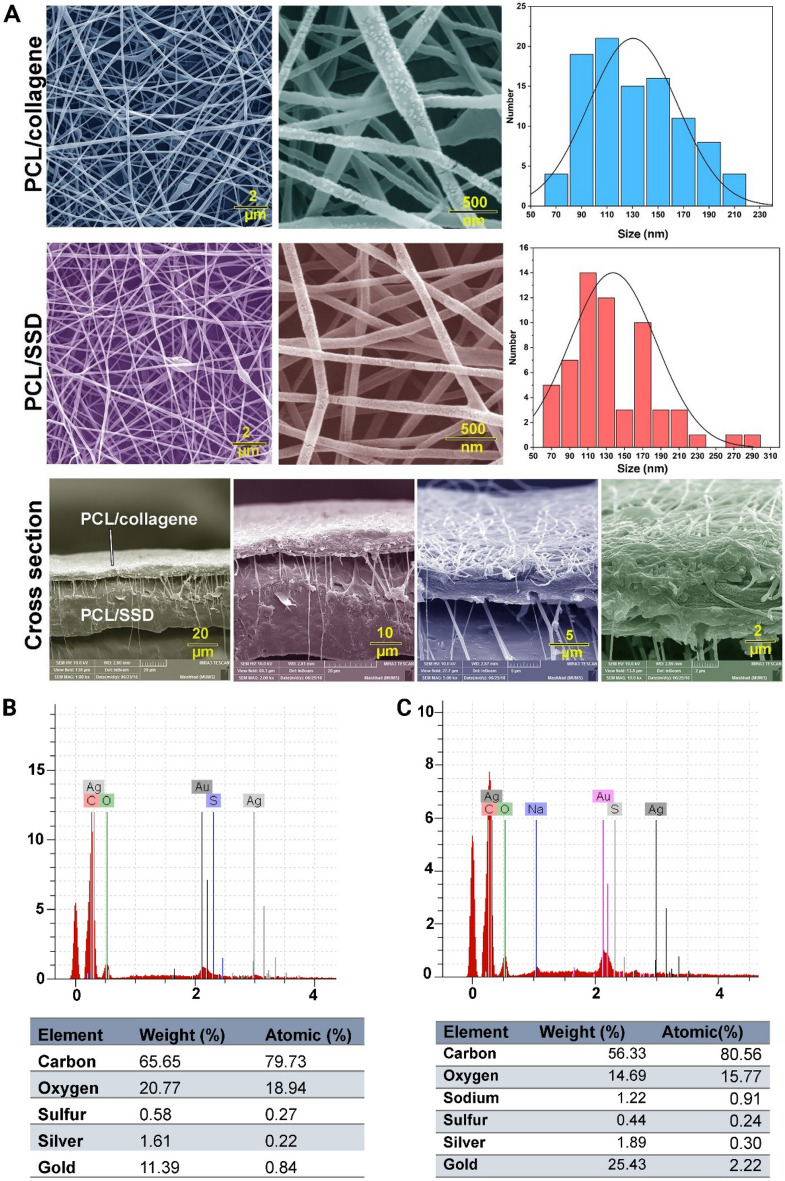
Morphological characteristics of the double-layered nanofibrous mat with FE-SEM. (**A**) the top layer is PCL/Coll and the bottom layer is PCL/SSD, which are shown with size distribution. The placement of the double-layered nanofibrous mat is shown in the bottom-most panel. The presence of nanofiber filaments in the cross-section between the two layers indicates that the two layers are connected to each other. Elemental analysis was done on the double-layered nanofibrous mat without GSE (**B**) and the double-layered nanofibrous mat with GSE (**C**).

The morphology, size of nanofibers, and elemental analysis were investigated through the SEM–EDX micrographs in the range of nanoscale (Fig. 2A–C). The diameters were calculated at 121.23 nm and 126.30 nm for PCL/SSD and PCL/Coll, respectively. The cross-section of the double-layered nanofibrous mat in which the (PCL/Coll) mat was superimposed to the PCL/SSD mat.

**Figure 3:**
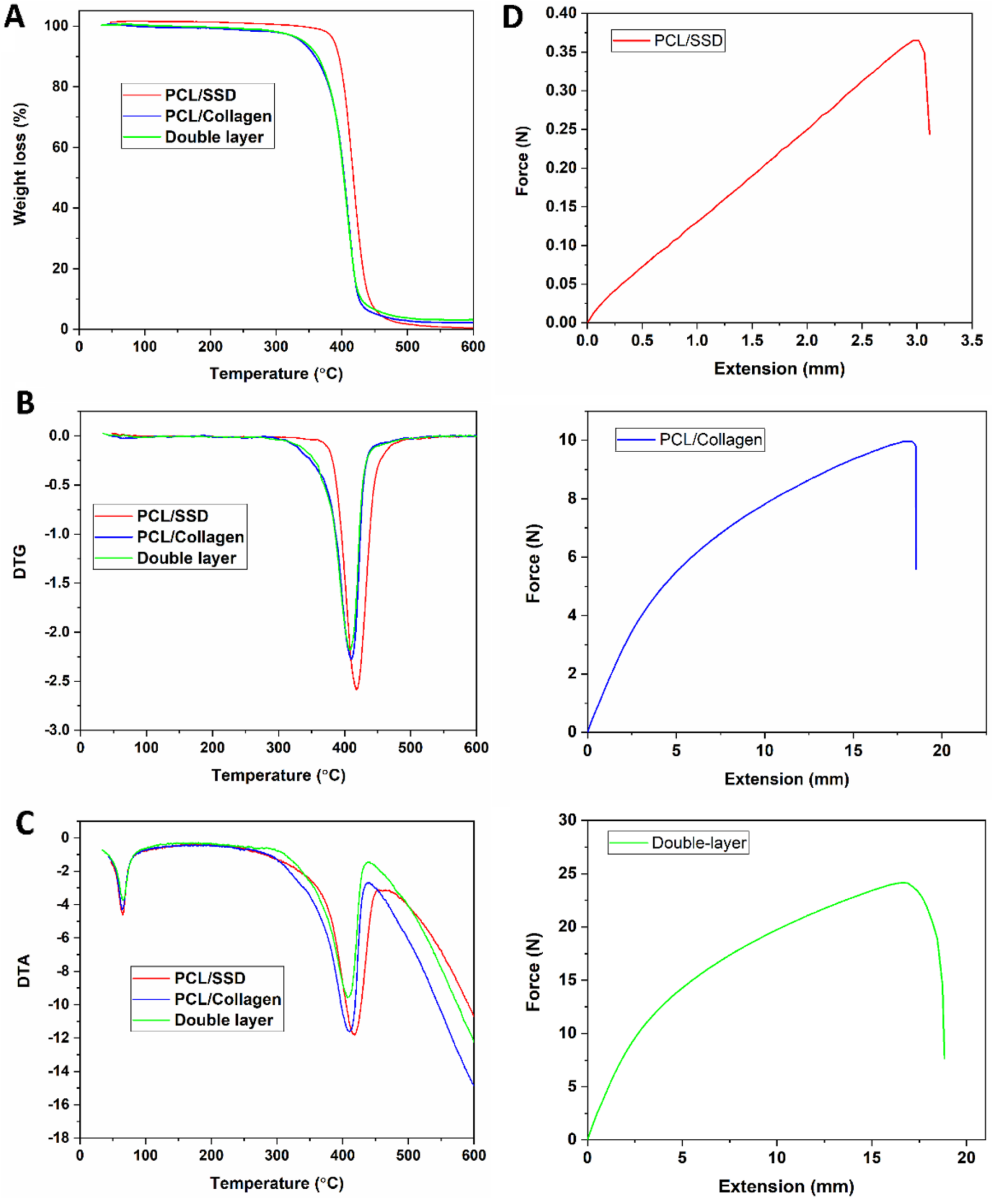
Thermal and mechanical properties of the electrospun nanofibers. (**A**) TGA, (**B**) DTG, and (**C**) DTA graphs of the samples. (**D**) Tensile test of the electrospun nanofibers.

Thermal properties of PCL/SSD, PCL/Coll, and the double-layered nanofibrous mat are shown in Fig. 3A–C. The primary decomposition temperature for PCL/SSD, PCL/Coll, and double-layered nanofibrous mat was around 400 °C, 350 °C, and 340 °C respectively. Young’s modulus of PCL/SSD, PCL/Coll, and double-layered nanofibrous membranes was 0.65, 2.3, and 13.6 MPa, respectively (Fig. 3D). These results were in the range of elastic modulus in normal human skin (i.e. 0.2–20 MPa), demonstrating suitable mechanical power and resistance for nanofibrous mat^26^.

### Release and antioxidant activity of the GSE

**Figure 4:**
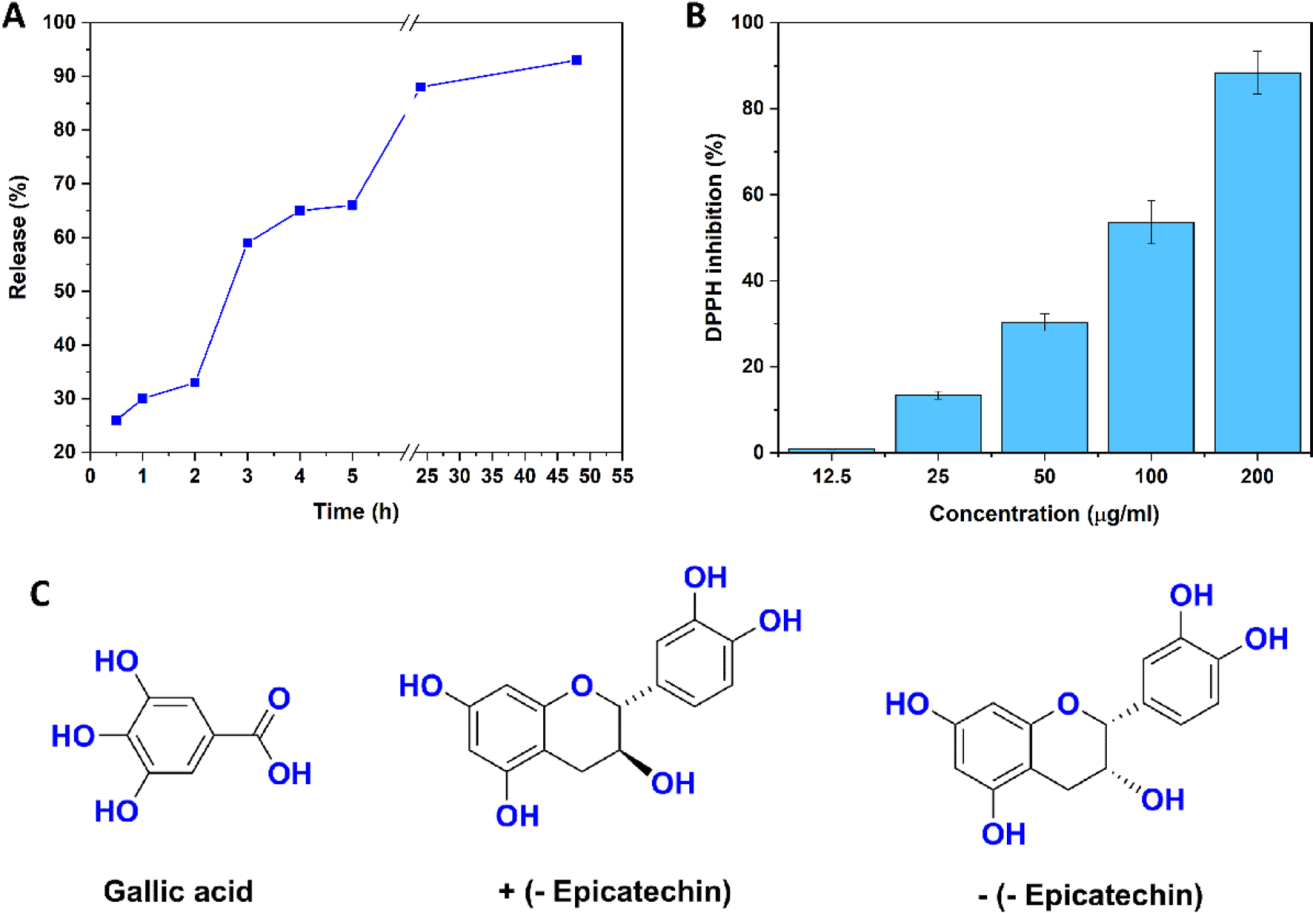
Release profile and antioxidant activity. (**A**) Release profile of the GSE from the nanofibrous mat. (**B**) Antioxidant activity of GSE. (**C**) Some available compounds responsible for antioxidant activity of the GSE.

The release study of the double-layered nanofibrous mat containing GSE is shown in Fig. 4A. The antioxidant property of GSE was investigated through the DPPH assay (Fig. 4B). As can be seen, the GSE showed antioxidant activity in a dose-dependent manner. GSE possesses a high content of flavonoids e.g., catechin, epicatechin, and some types of phenolic acids with active atom or transfer electron that are responsible for antioxidant activity^27^ as is shown in Fig. 4C. With increasing the concentration of the extract, the antioxidant properties of the extract also increased, and this shows that this extract has antioxidant properties. After 2 days 50 h, the scaffold released test measurement showed that the extract release percentage was approximately 90–95%, which is equivalent to 200 µg/ml of extract concentration.

### Antibacterial activity

**Figure 5:**
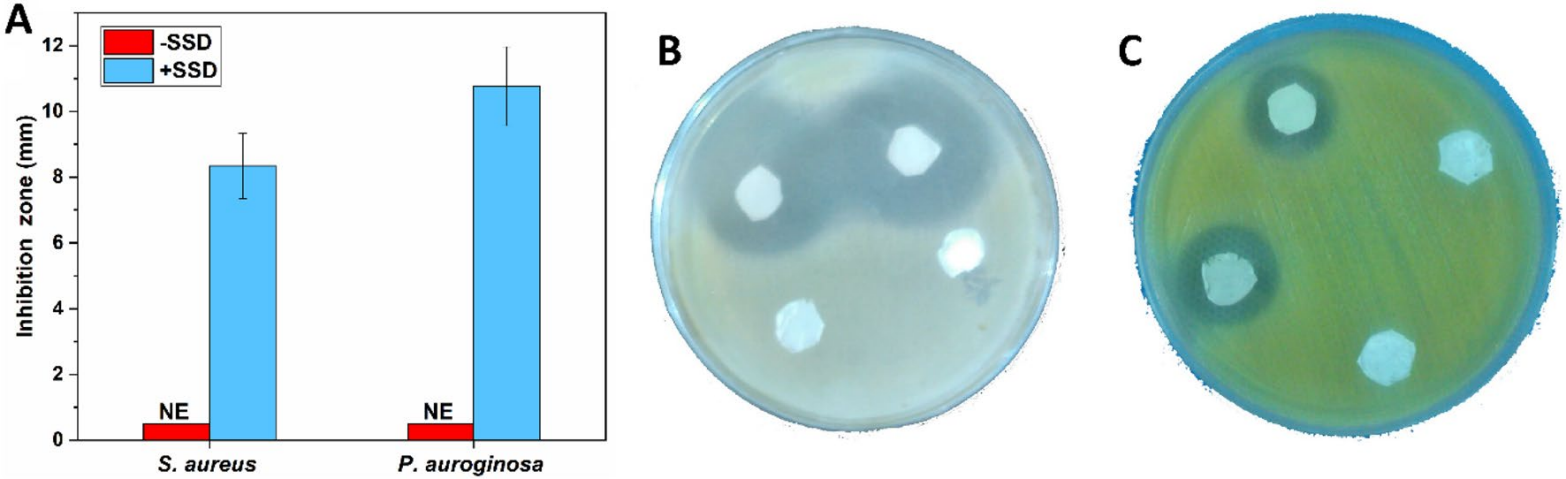
The antibacterial effect of the prepared nanofibrous mats against *S. aureus* and *P. aeruginosa* is shown in a bar graph (**A**). The inhibition zone against the nanofibrous mat with and without SSD in *S. aureus* (**B**) and *P. aeruginosa* is presented (**C**).

Antibacterial systems can enhance the tissue regeneration process by inhibiting pathogenic microorganisms^28,29^. The antibacterial activity of the double-layered nanofibrous mat with and without SSD against *S. aurous* and *P. aeruginosa* is shown in Fig. 5. The inhibition zone around the nanofiber sample shows the antibacterial effect of the SSD loaded in the nanofibrous mat. The nanofibrous mat exhibited higher activity against *P. aeruginosa*, compared to *S. aurous.*

### Cell proliferation, attachment, and migration

**Figure 6:**
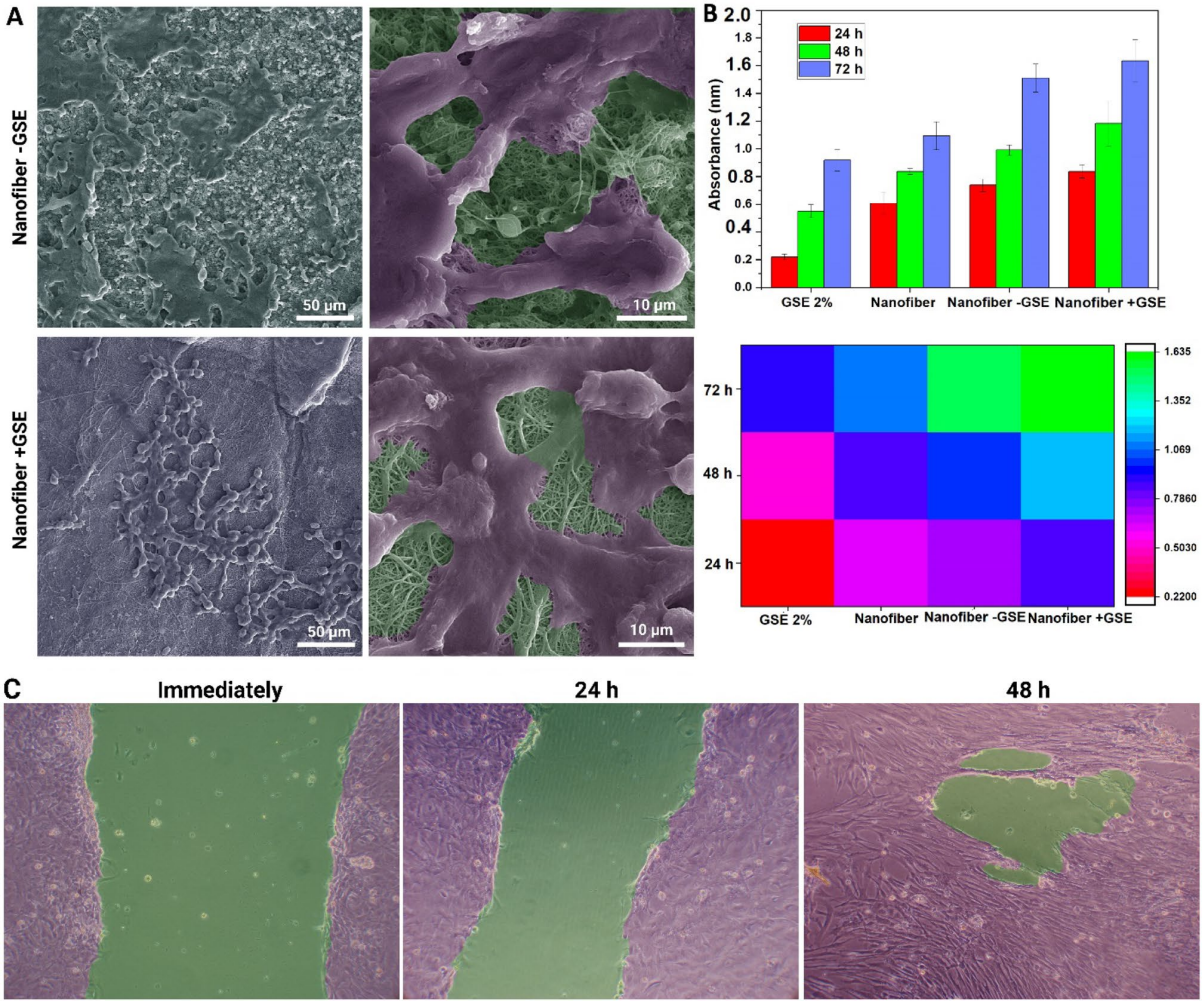
(**A**) FE-SEM image of HDF stem cells proliferation on the nanofibrous mat with and without GSE. The cells were artificially colored to be more distinguishable in the image. (**B**) Bar graph and heatmap related to the results of the MTT assay. (**C**) In vitro cell migration assay at different times. The images were colored to show the different areas.

The FE-SEM showed cell proliferation on the double-layered nanofibrous mat with and without GSE, and found that scaffolds provided a favorable environment (Fig. 6A). Statistical analysis of the data revealed that the double-layered nanofibrous mat had no effect on the viability of HDF stem cells after 48 h of culture (Fig. 6B). We discovered a high capacity for HDF stem cell adhesion and growth on the double-layered nanofibrous mat containing 2% GSE. The bright-field images of the cell migration assay and the scratch area immediately, 24, and 48 h after incubation with the nanofibrous mat. are shown in Fig. 6C. As can be seen, the gap (wound area) shrinks over time, indicating the nanofibrous mat's ability to facilitate cell migration**.**

### In vivo wound healing study

**Figure 7:**
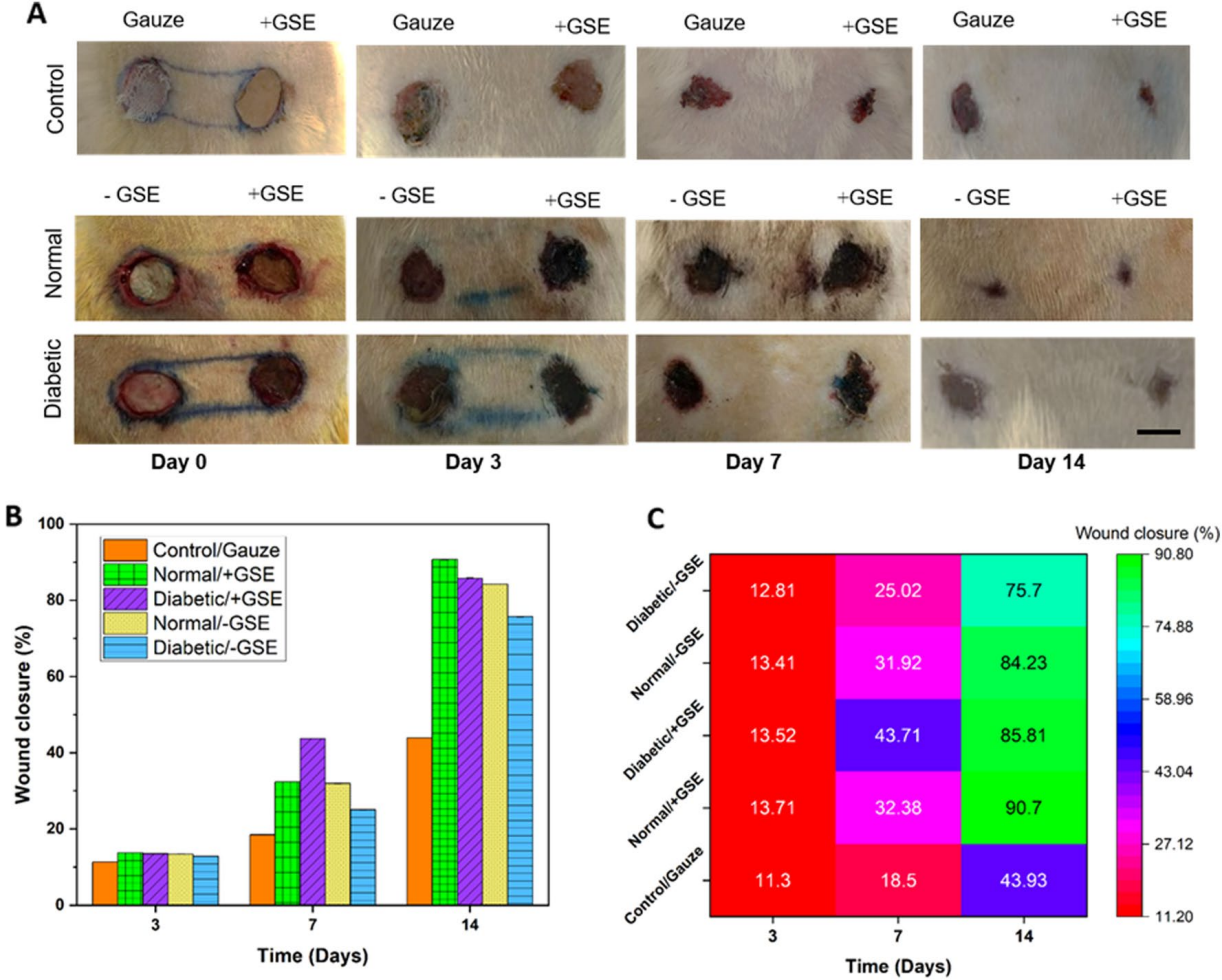
Macroscopic evaluation of wound healing in normal and diabetic rats. (**A**) Models on days 0, 3, 7, and 14 following surgery, demonstrating the wound's gradual healing over time. Scale bar represents 10 mm. (**B**) Wound closure percentage and heatmap graph in diabetic and normal groups with and without extract during days 3, 7 and 14 of the repair processes. (**C**) GSE represents treatment without grape seed extract; + GSE represents treatment with seed extract. Scale bar represents 10 mm.

Macroscopic images of the nanofibrous mats transplanted in rats in the diabetic, normal, and control groups during days 0, 3, 7, and 14 of the repair periods showed that the repair processes in the three groups were progressive (Fig. 7). During the healing process, no infection was seen at or around the transplant site in the transplanted groups according to macroscopic examination. Analysis of the process of wound closure in the normal and diabetic groups with and without GSE shows that in the normal group, transplantion of the nanofibrous mat with GSE in the right region of the rats looked more suitable than the left region, and the wound healing process is significantly faster compared with the control group with gauze. Wound closure in the two groups (normal and diabetic) with a double-layered nanofibrous mat with GSE was faster and better compared with the double-layered nanofibrous mat without GSE and the control group with gauze. However, the difference in wound closure between two sides of rats with and without GSE is not significant in the normal group macroscopically.

The wound healing in different groups was evaluated: in normal/ + GSE group, more than 90% wound closure was seen on the 14th day, while in the diabetic/ + GSE group, about 85.81% wound closure was observed. In normal/ + GSE group, wound closure completed (~ 90%) until day 14, while in normal/-GSE, diabetic/ + GSE, diabetic/-GSE, and control/gauze groups, it reached 84.23%, 85.81%, 75.7%, and 43.93%, respectively in this period of time. Therefore, dressing with the desired scaffolds accelerated the wound healing process.

**Figure 8:**
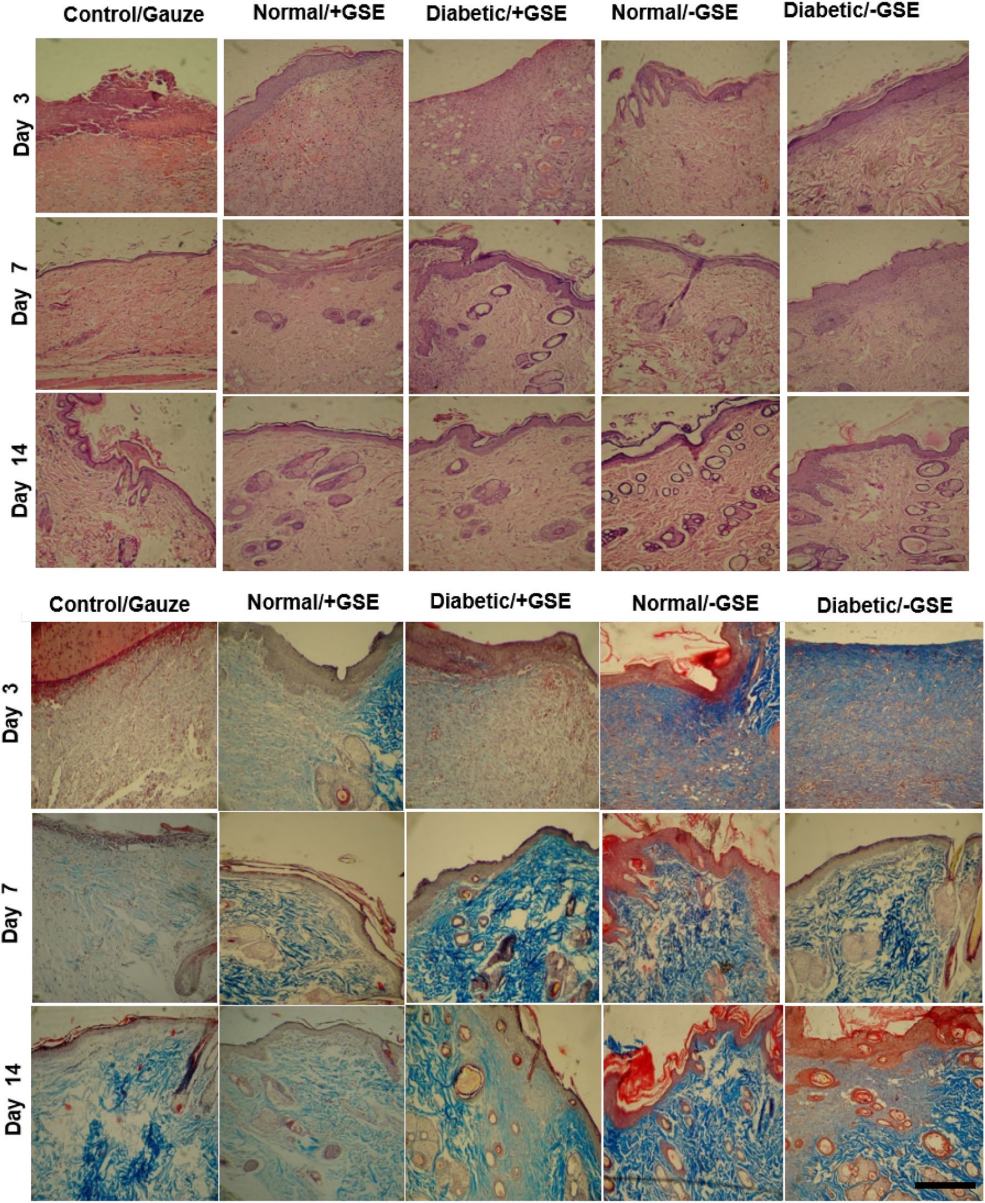
Microscopic evaluation of wound healing. Hematoxylin–eosin staining (**A**) and trichromassonʼs staining (**B**). Scale bar represents 200 µm.

Histological observations of hematoxylin–eosin staining by light microscopy on days 3, 7, and 14 were examined for collagen production, the epithelialization process, and skin appendage formations (Fig. 8A). The wound area for mats containing GSE in the normal group in 3,7, and 14 days was reduced compared to wounds sealed with mats alone in normal and diabetic rats.

The proliferation process in groups receiving GSE in normal and diabetic rats happened faster than groups without GSE at day 3. In groups with GSE, the presence of fibroblast cells and collagen fibers increased, compared to groups without GSE.

H& E staining shows the faster epidermal layer formation in wounds treated with the double-layered nanofibrous mat with GSE compared to the double-layered nanofibrous mat without GSE in diabetic and normal rats. After 14 days in the groups undergoing transplantion with double-layered nanofibrous mat containing GSE, the edges of the wound showed a good healing process towards the center of the wound. Clear granulation was seen immediately in the wound area after 3 days. Inflammatory cells such as lymphocytes and neutrophils were particularly reduced in the group receiving double-layered nanofibrous mat containing GSE. A uniform epithelium was seen in the entire wound area in all groups with double-layered nanofibrous mat on all days. After 14 days, new fibroblasts and collagen fibers were formed with special regulation and orientation under the new epithelium.

**Figure 9:**
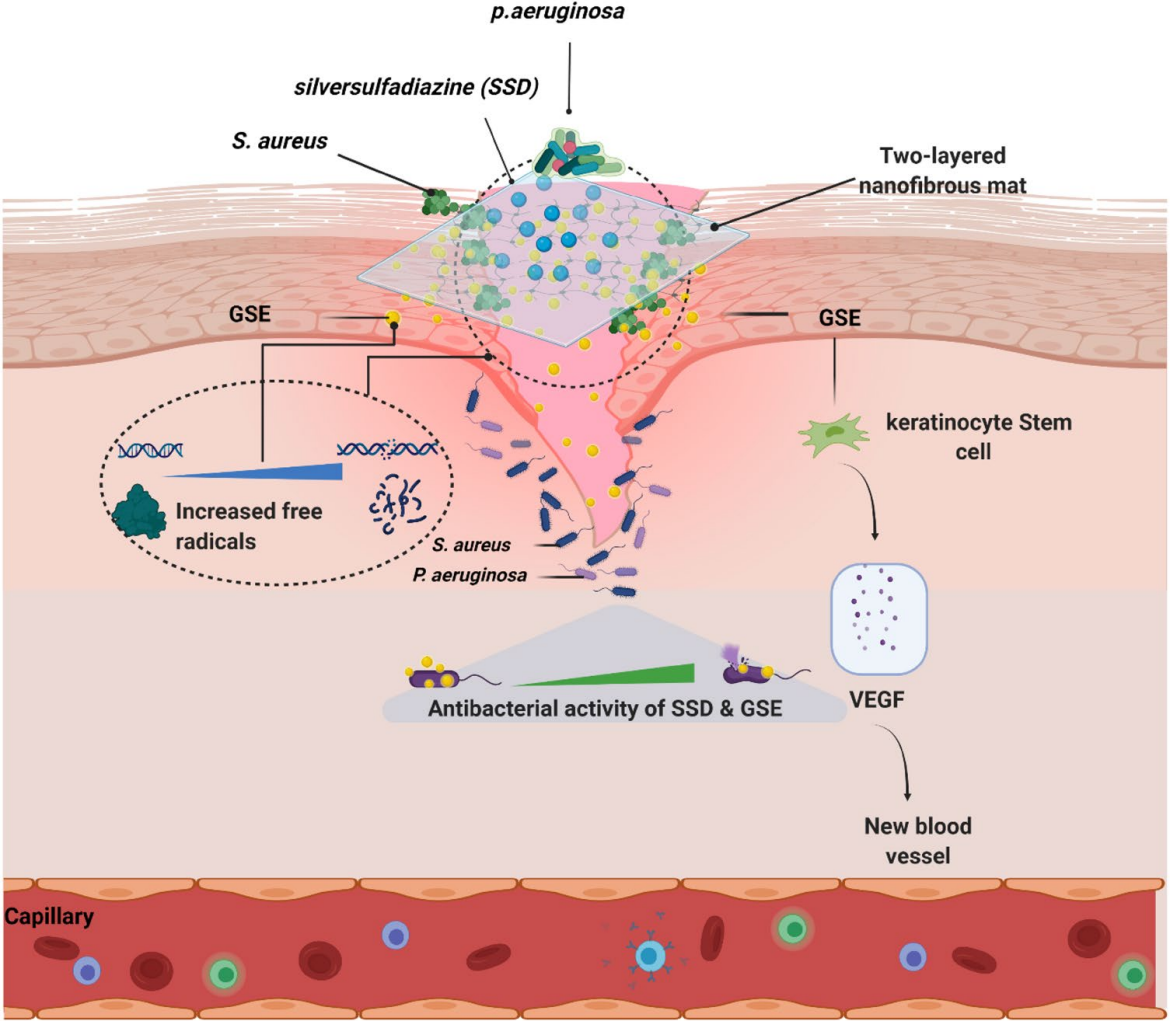
Schematic illustration of the application of a double-layered nanofibrous mat with antibacterial and antioxidant properties to promote wound healing.

On the other hand, the presence of a large number of matured hair follicles at the double –layered dressing treated wounds on day 14 demonstrated the effective healing ability (Fig. 8A). Masson's trichrome staining, which was used to evaluate collagen formation and remodeling, revealed a very clear difference between the nanofibrous mat with and without GSE (Fig. 8B). In normal and diabetic groups without GSE, collagen formation and maturation of fibers was in the early steps, so bundle formation did not occur properly. The development of collagen bundles was promoted in the double-layered nanofibrous mat without GSE when compared to the normal group, but the process of collagen formation was in earlier stages in the double-layered nanofibrous mat with GSE. Compared with other groups, the double-layered nanofibrous mat with GSE revealed final and formed collagen bundles with acceptable morphology, similar to normal skin. The collagen bundle shape and organization in the double-layered nanofibrous mat with GSE clearly demonstrated the formation of a high-quality skin that could serve as a flawless skin. Figure 9 shows the schematic illustration of the application of a double-layered nanofibrous mat with antibacterial and antioxidant properties to promote wound healing.

## Discussion

Electrospun wound dressings offer comfort and flexibility during and after application. Because there is no need for frequent changing of the dressing, the patient may be more adaptable. Due to their high compatibility with blood and tissues, biodegradable electrospun wound dressings promote the healing process and accelerate cell growth. The rate of tissue regeneration can be used to control the rate of scaffold degradation^30–34^.

Reduction of the wound healing period in diabetic patients is a major challenge for physicians due to the pathophysiology of these patients. Diabetic ulcer as a medical and economic problem has been observed around the world for many years, and the number of patients and the risk of amputation are constantly on the rise. One of the major concerns of diabetic wound care physicians is development of infection at the wound site, which leads to poor wound healing^35^. Due to these challenging conditions, the present study was conducted to develop methods in the field of reconstructive medicine with the aim of improving wound healing in normal and diabetic patients.

Strong antioxidant compounds such as proanthocyanidins and polyphenols that can facilitate wound healing are found abundantly in grape seed extract^36^. Proanthocyanidins are a group of biologically active polyphenolic bioflavonoids that are synthesized by many plants such as cranberry, blueberry, and grape seeds . The effect of proanthocyanidins in the body is 20 and 50 times higher compared with vitamins C and E, respectively. These antioxidant compounds prevent cell damage caused by the production of free radicals during wound healing and neutralize the effects of free radicals^37^**.** Several studies either on animal models or humans have shown the ability of GSE to promote wound healing^38,39^.

However, GSE loading in nanofiber scaffolds has not been used to evaluate the wound healing process. Therefore, in this study, in the first stage, we designed a double-layered nanofibrous mat the top layer of which was PCL/Coll, causing fibroblast cells to have better adhesion and migration to its surface in the in vivo environment^40^. The bottom layer contains silver sulfadiazine, which has antibacterial properties^41^. These two layers were then exposed to 2%GSE as an antioxidant agent.

The double-layered nanofibrous mat was initially evaluated for mechanical properties. In terms of appearance, the formed scaffold resembled nanofibers, and the two layers were placed well on top of each other with interaction, as evidenced by SEM results. FE-EDX results also showed the presence of silver ions as an indicator of the presence of silver sulfadiazine in the nanofibrous mat^20^. The presence of sodium and sulfur ions also indicates the presence of GSE in the scaffold^42^.

TGA results also showed the degradation properties of the double-layered nanofibrous mat in comparison with monolayer of nanofiber. The mechanical properties of double-layered nanofibrous mat were more powerful than those of the mono-layered one, which shows the uniformity and formation of the double-layered nanofibrous mat.

Antibacterial materials are of paramount importance in bacterial infection therapy^43,44^. Some studies have shown that PRP has antibacterial properties and leads to wound healing by reducing bacterial colonies and cell inflammation in infected and non-infected excision wounds^45^.

According to previous studies, some antibacterial mechanisms have been proposed for GSE. In summary, hydrophobic phenolic components interact with bacterial surface structures and lipopolysaccharides, leading to a decrease in membrane stability. GSE has an inhibitory influence against a wide range of Gram-negative and Gram-positive bacteria^46,47^. Of course, the exact mechanisms of GSE compounds during the wound healing process cannot be confirmed in this work, but we may make suggestions in connection with other reports^46,48^. Burst release from the scaffold during two days with a concentration 200 µg/ml reached 90% of the antioxidant properties. It is quickly released from the scaffold within two days to prevent infection in the initial stages of wound healing. According to previous studies, this concentration of GSE did not have a negative effect on the growth of fibroblast cells^49^. Given the hypothesis that oxidative stress is present in wound healing, GSE seems to be a powerful antioxidant that is able to eliminate the reactive oxygen species (ROS), leading to a faster healing process in wound healing. When skin is damaged, huge levels of ROS are created during the inflammatory phase of wound healing, causing biological damage such as degradation of lipid, protein, and nucleic acid as well as cell death, which impairs the wound healing process^46^. The use of antioxidants can effectively help enzymatic healing and improve metabolism in the inflammatory stage of wound healing.

The mechanism of SSD takes place through three different ways. (1) Production of active oxygen species derived from soluble oxygen from the catalytic activity area. (2) Cross-linking with silver in the position of hydrogen bonds between two strands of DNA (3) Inhibition of enzyme activity through intracellular silver ions. During the investigation of agents containing silver, SD is used rather than other different compounds due to its broad antibacterial effects^14^. However, the purpose of the current study was not to investigate this mechanism of action, and previous studies could be referred to for this purpose. The antibacterial properties of silver sulfadiazine used in the scaffold were well demonstrated by the formation of an inhibition zone around the scaffold. Loading the SSD in the lower layer of the double-layered nanofibrous mat makes it possible to prevent infection during the healing period by slowly releasing it into the surrounding environment. This has been evaluated and investigated in a previous study on this drug release from the nanofibrous mat. Release profile demonstrated a primarily high release throughout the first 5 days, tracked by a more continuous and gradual release until day 20^18^.

The adhesion of fibroblast cells to the surface of the scaffold indicates that the components used were not toxic to these cells^20,50^. After two days, the scratch into the flask containing fibroblast cells was likewise filled and repaired.

A nanofibrous mat with these properties and characterization was applied as a wound dressing on the rat model of full thickness skin wound. The healing process was followed for 3, 7, and 14 days. Macroscopic observation revealed that wounds treated with double-layered nanofibrous mat containing GSE in both normal and diabetic groups heal more effectively in terms of appearance on days 3, 7, and 14. H&E routine staining of healed tissue after 14 days revealed no scarring in the wound area in GSE receiving groups of normal and diabetic rats. The percentage of wound contraction was as follows: Normal/ + GES > Diabetic/ + GSE > Normal/-GES > Diabetic/-GSE > gauze/control.

In accordance with previous studies on chitosan-capped derivative^13^, platelet–rich plasma^45^, biomimetic LBL structured nanofibrous matrices^51^, LBL modified nanofibrous mats^52^, and silver/kaolinite nanocomposite ^53^, the new dressing fabricated in this study could repair the wound healing process (within 14 days) without causing scars or infection. The present study, however, was different from the aforementioned studies in that the designed scaffold was applied for wound healing in rats that had diabetes in this study.

Despite its major strengths, this study had some limitations that future studies may take into account. Since powdered grape seed extract is soluble in ethanol and the solvent of PCL cannot dissolve the grape seed extract, we were not able to electrospun the extract powder in this study. Therefore, a small proportion of the extract was exposed to the wound, yet this small proportion was also effective in promoting wound healing.

Because this was an animal study, clinical trials are needed as the next step to approve the impact of double-layered nanofibrous mats with grape seed extract on wound healing in humans. In the current study, grape seed extract was used only once before implanting the nanofibrous mat, but for further examination, it is necessary to examine its effect at different time intervals.

Since one dose of grape seeded extract was applied in this study, future studies are recommended to use higher doses of the grape seed extract.

It is worth mentioning that if the grape seed extract is used together with a hydrogel scaffold, it may show better results in vivo, which is the topic our research team is currently working on.

## Conclusion

In this study, PCL was chosen as a cost-effective, non-toxic, and biodegradable material for the fabrication of nano/microfibrous scaffolds. Our findings shed light not only on the design of a PCL/SSD, PCL/Coll nano/microfibrous matrix with good adhesiveness, increased porosity, and supportive elasticity and stiffness for stem cell lineage commitment, but also on the therapeutic applications of this nanofibrous mat for wound healing and skin reconstitution. Overall, the main stages of healing were clearly shortened in grafted wounds by double-layered nanofibrous mat, with the transition from inflammation to proliferation and remodeling becoming shorter. As a result, this blended mat could be a candidate for faster and more effective skin tissue repair and remodeling. Of course, association of GSE with such preparation may help us to introduce a novel formulation for variety of wounds e.g., diabetic wounds as shown in this work. To accelerate wound healing, more research is needed to assess the effect of nanofibrous mat thickness as well as different time points over a long period of time.
